# Phylogenetics and Pathogenesis of Early Avian Influenza Viruses (H5N1), Nigeria

**DOI:** 10.3201/eid1411.080557

**Published:** 2008-11

**Authors:** Comfort O. Aiki-Raji, Patricia V. Aguilar, Yong-Kuk Kwon, Sue Goetz, David L. Suarez, Aashish I. Jethra, Oyekanmi Nash, Christopher A.O. Adeyefa, Festus D. Adu, David Swayne, Christopher F. Basler

**Affiliations:** University of Ibadan, Ibadan, Nigeria (C.O. Aiki-Raji, O. Nash, C.A.O. Adeyefa, F.D. Adu); Mount Sinai School of Medicine, New York, New York, USA (P.V. Aguilar, A.I. Jethra, C.F. Basler); US Department of Agriculture, Athens, Georgia, USA (Y.-K. Kwon, S. Goetz, D.L. Suarez, D. Swayne); 1 These authors contributed equally to this work.

**Keywords:** Africa, H5N1, influenza, Nigeria, pathotyping, phylogenetic analysis, dispatch

## Abstract

Three highly pathogenic avian influenza subtype H5N1 and 4 Newcastle disease viruses were isolated from sick or dead chickens in southwestern Nigeria. Sequencing and phylogenetic analysis placed them within H5N1 subclade 2.2.2. Intravenous and intranasal pathogenicity tests produced systemic disease with vascular endothelial cell tropism in chickens.

The first official report of avian influenza virus (H5N1) in Africa was made in January 2006 (*1*). Before then, surveillance was ongoing at the poultry clinic of the University of Ibadan Veterinary Teaching Hospital to identify causes of death in chickens in Nigeria.

## The Study

Nasopharyngeal and cloacal swab samples were collected from sick and dead birds found on farms near Ibadan, Nigeria, and injected into 10-day-old embryonating chicken eggs. Retrospective analysis of isolates obtained in or near January 2006 identified influenza virus (H5N1) in 3 samples. In addition, 4 Newcastle disease virus isolates were obtained, which highlights the cocirculation of Newcastle disease virus and influenza viruses (H5N1) in Nigerian poultry and emphasizes the need for specific virologic testing to distinguish the clinically similar poultry diseases caused by these 2 pathogens.

The 3 avian influenza (H5) isolates were designated influenza A/chicken/Nigeria/228-5/2005, A/chicken/Nigeria/228-6/2006, and A/chicken/Nigeria/228-10/2006. Nucleotide sequences of the coding regions of all 8 segments of the 3 viruses demonstrated that all isolates possessed a multiple basic amino acid at the hemagglutinin (HA) cleavage site with the sequence PQGERRRKKR. Sequences at this site were identical to those of highly pathogenic avian influenza (HPAI) subtype H5N1 viruses from Europe, Russia, Asia, and from recent isolates from the Lagos state of Nigeria (*2*); it lacks a single basic residue when compared with HA from strains from southeastern People’s Republic of China, Vietnam, Cambodia (PQRERRRKKRG), and Thailand (PQREKRRKKRG) (*3*). Other notable features of the sequences were the absence of the H274Y genetic change associated with high-level resistance to oseltamivir in influenza neuraminidase 1 (N1) viruses (*4*). Similarly, known amantadine resistance–linked mutations were absent. The nonstructural (NS) 1 open reading frame encodes a 5-aa deletion at positions 80–84, as has been observed since 2001 in subtype H5N1 isolates from poultry. One of the isolates (A/chicken/Nigeria/228-10/2006) also has a C-terminal amino-acid extension, which is predicted to affect the function of the PDZ-ligand domain otherwise present at the C terminus of the NS1 protein (*5*,*6*). This sequence change did not detectably affect the ability of NS1 to block interferon induction when expressed transiently in 293T cells (data not shown). The polymerase basic 2 protein (PB2) of these viruses possesses a lysine residue at position 627, an amino acid previously implicated in mammalian adaptation of subtype H5N1 viruses (*7*–*9*).

Phylogenetic analysis based on the HA sequence and on complete genome sequences of HPAI (H5N1) strains grouped the 3 new isolates from Nigeria with other isolates from Europe, the Middle East, and Lagos state of Nigeria. According to recent classification by the H5N1 Evolution Working Group, the viruses belong to clade 2.2.2 (previously referred to as the European-Middle Eastern-African clade 1) (*10*). The Technical Appendix, shows the phylogenetic trees generated on the basis of the sequences of HA, NA, nucleocapsid protein (NP), and NS segments. On the basis of phylogenetic analyses of all 8 segments, no evidence of reassortment was observed among the newly sequenced HPAI isolates from Nigeria. Although prior reports (*2*,*3*,*11*) have suggested 3 independent introductions of HPAI viruses into Nigeria, our analysis of viruses in this study and in GenBank identified only 2 clades (clades 2.2.2 and 2.2.3) among the Nigeria isolates, suggesting 2 unique introductions into Nigeria.

The virulence of the HPAI virus isolated in poultry was assessed by intravenous injection of a 1:10 dilution of allantoic fluid into groups of 4-week-old SPF White Leghorn chickens, 8 per group, as previously described (*12*). According to results of this assay, all 3 isolates were highly pathogenic (Table); mean times to death were 1.0, 1.3, and 1.4 days, similar to times previously reported for other European-Asian lineage HPAI (H5N1) viruses (*13*).

**Table:** Virus isolation titers from chickens inoculated with highly pathogenic influenza A/chicken/Nigeria/228-5/2005 (H5N1) and controls*

Sample	Virus-positive swabs/swabs tested, no. (mean titer† as log_10_ EID_50_/mL)			Virus-positive tissues/tissues tested, no. (mean titer† as log_10_ EID_50_/g)				
	Oropharyngeal	Tracheal		Heart	Kidney	Lung	Muscle	Brain
Sham	0/2	0/2		0/2	0/2	0/2	0/2	0/2
Experimental								
1 dpi	2/2 (2.4)	1/2 (1.7)		1/2 (2.9)	1/2 (4.5)	1/2 (4.9)	1/2(3.9)	1/2 (3.5)
2 dpi	2/2 (6.1)	2/2 (6.8)		2/2 (8.7)	2/2 (7.9)	2/2 (7.6)	2/2 (6.8)	2/2 (5.6)

*Virus isolated from tissues and swabs sample on 1 and 2 days postinoculation (dpi). EID_50_, mean embryo infectious doses. †Limit of detection <10^1.9^ EID_50_/g of tissue or <10^0.9^ EID_50_/mL sample.

To determine infectivity and pathogenicity, using a simulated natural respiratory route of exposure, we intranasally inoculated ten 4-week-old White Leghorn chickens with strain A/chicken/Nigeria/228-5/2005 (10^6^ mean embryo infectious doses). The caged birds were inspected daily for clinical signs and death. Two chickens that were not inoculated and were maintained as negative controls were euthanized (100 mg sodium pentobarbital/kg body weight) at 0 days postinoculation (dpi). At 1 dpi, 2 of the injected birds were euthanized. On 2 dpi, postmortem examinations were performed on 2 birds that had died, and samples were collected for virus isolation and for histopathologic and immunohistochemical examination of a variety of tissues. For immunohistochemical examination, a monoclonal antibody against influenza A nucleoprotein was used as previously described (*14*). Virus isolation and titration were conducted with brain, heart, lung, kidney, breast muscle, oropharyngeal, and cloacal samples; embryonating chicken eggs were used (*12*).

After intranasal inoculation with A/chicken/Nigeria/228-5/2005, all birds died or were euthanized for severe illness within 3 dpi (mean time to death 2.125 days). At 1 dpi, both intranasally inoculated chickens sampled were infected, as evidenced by low virus titers in oropharyngeal swabs; however, only 1 chicken had systemic infection with moderate titers of virus in tissues (Table). Histologically, both chickens lacked lesions, but the spleen of 1 chicken had a few avian influenza virus–positive histiocytes. The intranasally inoculated chickens that died and were necropsied on 2 dpi had exhibited mild to moderate listlessness, a hunched posture, and partial closure of eyelids a few hours before death. Gross lesions included scattered petechia in epicardial fat within the coronary groove (Figure, panel **A**), a few petechia in mucosa of the ventriculi (gizzards), slight increase in pericardial fluid, and swollen kidneys. Swabs from respiratory and alimentary tracts and the tissues had high titers of the virus (Table). Histologically, tissues with lesions coincided with sites of virus replication and indicated severe systemic infection. The most severe lesions were severe interstitial pneumonia with edema (Figure, panel **B**), moderate to severe myocyte degeneration and necrosis in the heart, moderate nonsuppurative encephalitis, moderate necrotizing rhinitis, moderate lymphohistiocytic depletion and apoptosis in the spleen, and mild to moderate degeneration and necrosis of pancreatic acinar epithelium (Figure, panel **C**). Scattered lesions of necrosis and inflammation were seen in liver, cloacal bursa, thymus, proventriculus, ventriculus, and pancreatic islets. Influenza A virus was localized to necrotic cells, which most frequently included blood vessel endothelium throughout the body (Figure, panel **D**), cardiac myocytes (Figure, panel **E**), pulmonary histiocytes and heterophils (Figure, panel **D**), neurons and glial cells in the brain, splenic histiocytes and cellular debris, and renal tubular epithelium. Virus was less frequently identified in Kupffer cells and hepatocytes; histiocytes in lamina propria of alimentary tract, thymus, and cloacal bursa; pancreatic acinar and islet epithelium (Figure, panel **F**); proventricular epithelium; and bone marrow myeloid cells.

**Figure:**
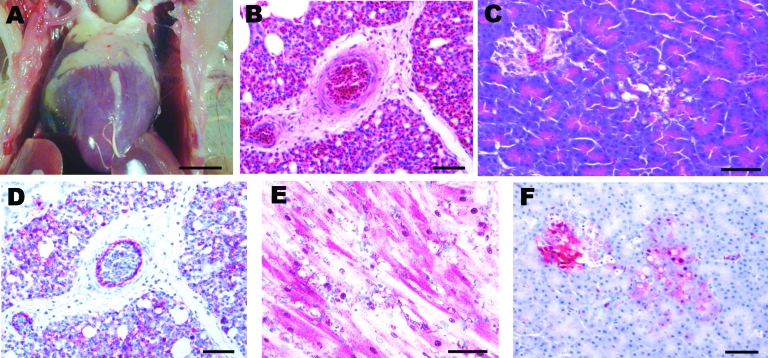
Experimental studies in chickens inoculated intranasally with highly pathogenic influenza A/chicken/Nigeria/228-5/2005 (H5N1) and sampled 2 days postinoculation. A) Photograph (scale bar = 1 cm) of a gross lesion, showing petechia in epicardial fat in the coronary groove. B,C) Photomicrographs (scale bars = 50 μm) of tissue sections stained with hematoxylin and eosin, showing severe histiocytic and heterophilic interstitial pneumonia with moderately interlobular edema (panel B) and pancreatic acinar epithelium (panel C). D–F) Photomicrographs (scale bars = 50 μm) of tissue sections stained immunohistochemically to demonstrate avian influenza virus nucleoprotein, showing avian influenza virus antigen in pulmonary and blood vessel endothelial cells and histiocytes (panel D), nuclei and cytoplasm of cardiac myocytes (panel E), and pancreatic acinar epithelium and islet cells (panel F).

## Conclusions

The 3 early HPAI (H5N1) isolates from Nigeria, which belonged to clade 2.2.2, produced in chickens a systemic infection characterized by virus replication and associated necrotic and inflammatory lesions in critical internal organs such as the heart, brain, and lungs. A prominent vascular tropism of the virus was evidenced by widespread replication in blood vessel endothelium throughout the body and is typical of other HPAI viruses (H5N1) of the Asian lineage.

## Supplementary Material

Technical Appendix Phylogenetics and Pathogenesis of Early Avian Influenza Viruses (H5N1), Nigeria
