# Supplementary material for: Phylogenetics and Pathogenesis of Early Avian Influenza Viruses (H5N1), Nigeria
Source: Emerg Infect Dis. 2008 Nov;14(11):1753–5. doi: 10.3201/eid1411.080557 (PMC2630749; doi:10.3201/eid1411.080557)
Supplement: Technical Appendix — Phylogenetics and Pathogenesis of Early Avian Influenza Viruses (H5N1), Nigeria [file 08-0557_Techapp-s1.pdf]

Phylogenetics and Pathogenesis of Early Avian Influenza Viruses (H5N1), Nigeria

Technical Appendix

Neighbor-joining phylogenetic trees shown on the following pages were generated based on the A) hemagglutinin, B) neuraminidase, C) nucleocapsid protein, and D) nonstructural sequences of influenza viruses (H5N1). Influenza (H5N1) clades (2.2.1, 2.2.2 or 2.2.3) are indicated on trees. Viruses sequenced in this study are shown in **boldface**. Numbers indicate bootstrap values. Complete nucleotide sequences were aligned by using the Clustal program (*1*), and phylogenetic analyses were performed by using the PAUP program, version 4.0 beta 10 (*2*), with the neighbor-joining method using the HKY85 distance formula. Bootstrap analyses were conducted with 1,000 to place confidence values on groupings within trees (*3*).

References

1. Larkin MA, Blackshields G, Brown NP, Chenna R, McGettigan PA, McWilliam H, et al. ClustalW and ClustalX version 2. Bioinformatics. 2007;23:2947–8. PubMed DOI: 10.1093/bioinformatics/btm404

2. Swofford DL. PAUP* (Phylogenetic analysis using parsimony) (* and other methods), version 4. Sunderland (MA): Sinauer Associates; 2002.

3. Felsenstein J. Confidence limits on phylogenies: an approach using the bootstrap. Evolution. 1985;39:783–91. DOI: 10.2307/2408678

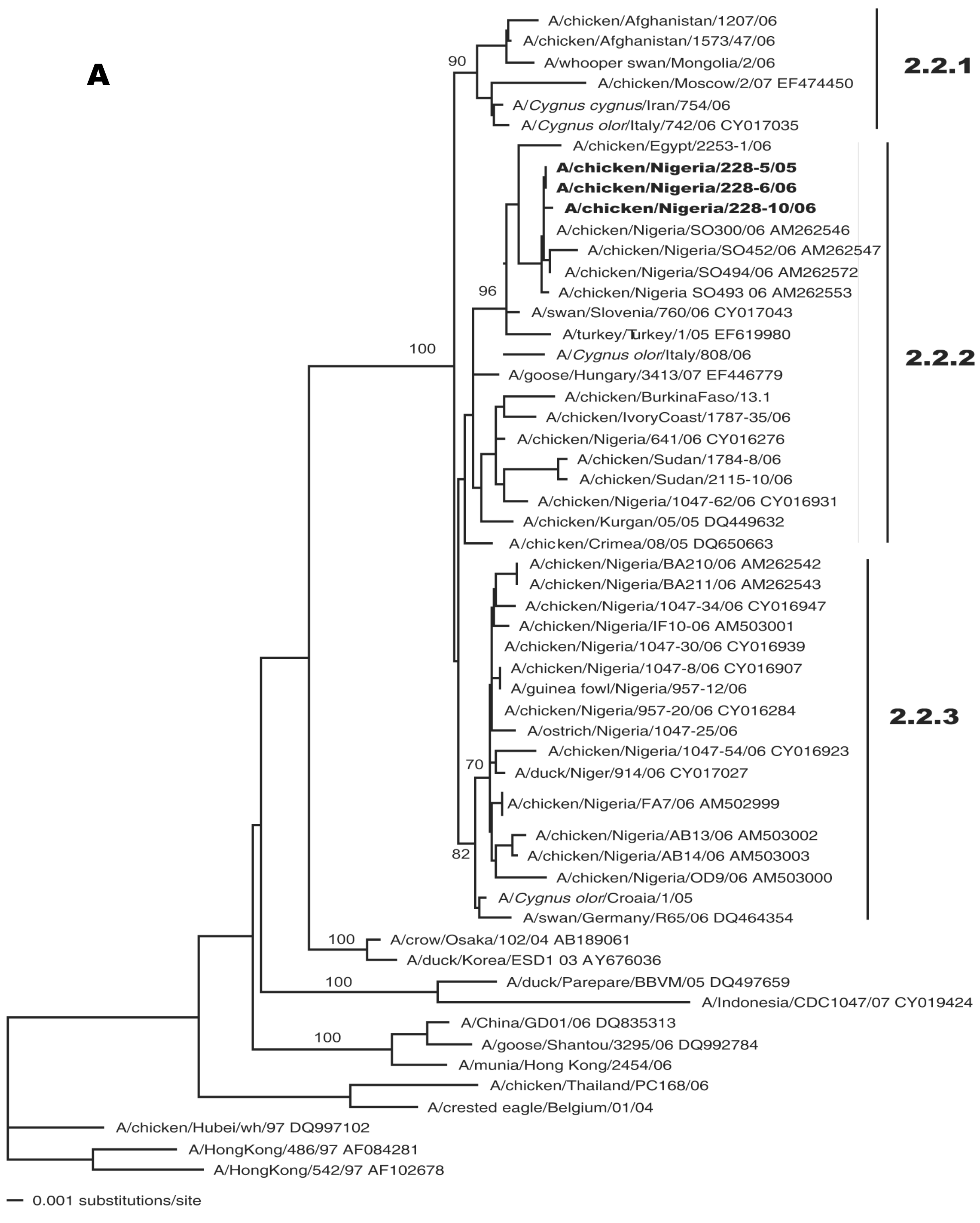

A
A/chicken/Afghanistan/1207/06
A/chicken/Afghanistan/1573/47/06
A/whooper swan/Mongolia/2/06
A/chicken/Moscow/2/07 EF474450
A/Cygnus cygnus/Iran/754/06
A/Cygnus olor/Italy/742/06 CY017035
2.2.1
90
A/chicken/Egypt/2253-1/06
A/chicken/Nigeria/228-5/05
A/chicken/Nigeria/228-6/06
A/chicken/Nigeria/228-10/06
A/chicken/Nigeria/SO300/06 AM262546
A/chicken/Nigeria/SO452/06 AM262547
A/chicken/Nigeria/SO494/06 AM262572
A/chicken/Nigeria SO493 06 AM262553
96
A/swan/Slovenia/760/06 CY017043
A/turkey/Turkey/1/05 EF619980
100
A/Cygnus olor/Italy/808/06
A/goose/Hungary/3413/07 EF446779
2.2.2
A/chicken/BurkinaFaso/13.1
A/chicken/IvoryCoast/1787-35/06
A/chicken/Nigeria/641/06 CY016276
A/chicken/Sudan/1784-8/06
A/chicken/Sudan/2115-10/06
A/chicken/Nigeria/1047-62/06 CY016931
A/chicken/Kurgan/05/05 DQ449632
A/chicken/Crimea/08/05 DQ650663
A/chicken/Nigeria/BA210/06 AM262542
A/chicken/Nigeria/BA211/06 AM262543
A/chicken/Nigeria/1047-34/06 CY016947
A/chicken/Nigeria/IF10-06 AM503001
A/chicken/Nigeria/1047-30/06 CY016939
A/chicken/Nigeria/1047-8/06 CY016907
A/guinea fowl/Nigeria/957-12/06
A/chicken/Nigeria/957-20/06 CY016284
A/ostrich/Nigeria/1047-25/06
2.2.3
A/chicken/Nigeria/1047-54/06 CY016923
70
A/duck/Niger/914/06 CY017027
A/chicken/Nigeria/FA7/06 AM502999
A/chicken/Nigeria/AB13/06 AM503002
82
A/chicken/Nigeria/AB14/06 AM503003
A/chicken/Nigeria/OD9/06 AM503000
A/Cygnus olor/Croaia/1/05
A/swan/Germany/R65/06 DQ464354
100
A/crow/Osaka/102/04 AB189061
A/duck/Korea/ESD1 03 AY676036
100
A/duck/Parepare/BBVM/05 DQ497659
A/Indonesia/CDC1047/07 CY019424
A/China/GD01/06 DQ835313
100
A/goose/Shantou/3295/06 DQ992784
A/munia/Hong Kong/2454/06
A/chicken/Thailand/PC168/06
A/crested eagle/Belgium/01/04
A/chicken/Hubei/wh/97 DQ997102
A/HongKong/486/97 AF084281
A/HongKong/542/97 AF102678
0.001 substitutions/site

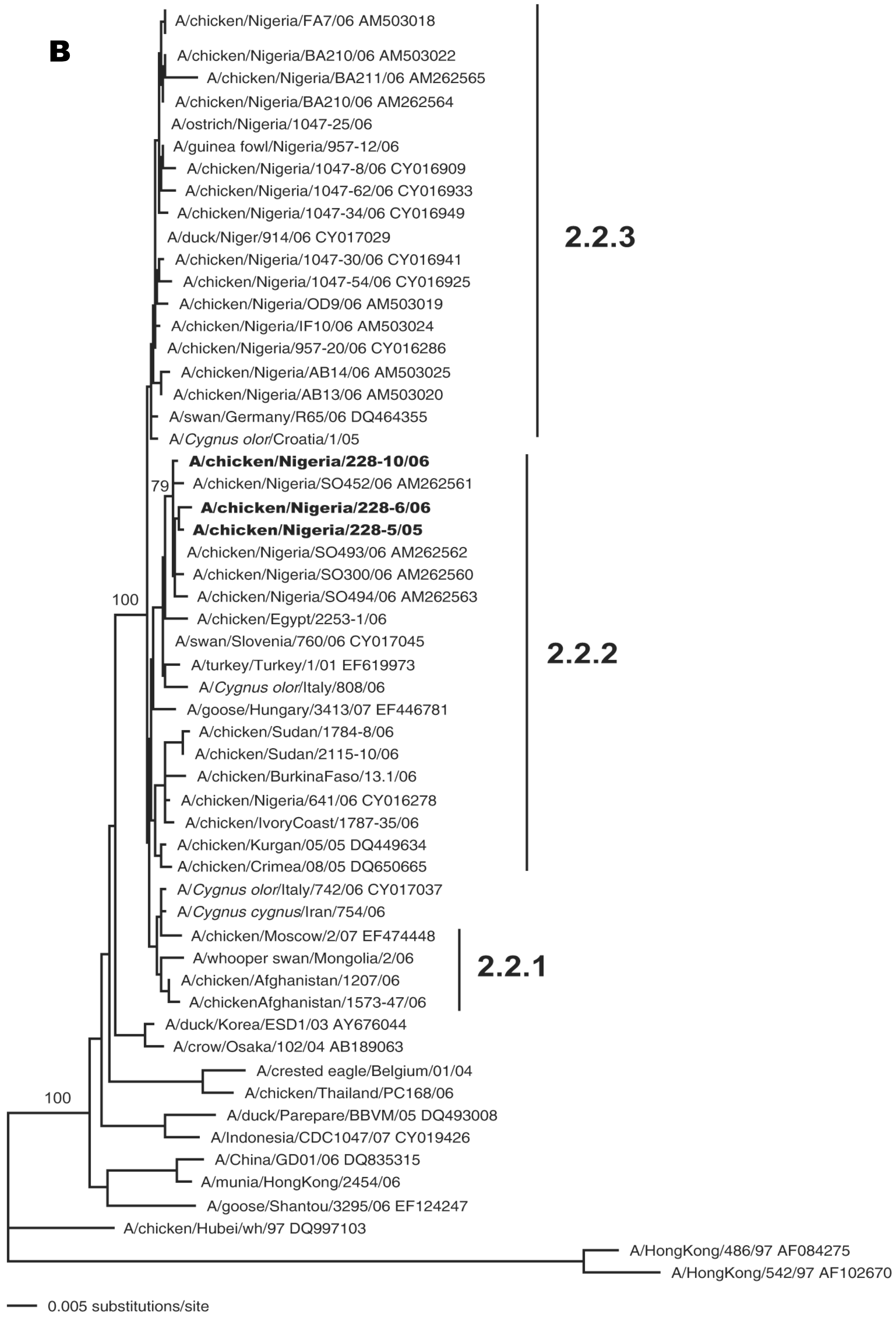
B
A/chicken/Nigeria/FA7/06 AM503018
A/chicken/Nigeria/BA210/06 AM503022
A/chicken/Nigeria/BA211/06 AM262565
A/chicken/Nigeria/BA210/06 AM262564
A/ostrich/Nigeria/1047-25/06
A/guinea fowl/Nigeria/957-12/06
A/chicken/Nigeria/1047-8/06 CY016909
A/chicken/Nigeria/1047-62/06 CY016933
A/chicken/Nigeria/1047-34/06 CY016949
A/duck/Niger/914/06 CY017029
A/chicken/Nigeria/1047-30/06 CY016941
A/chicken/Nigeria/1047-54/06 CY016925
A/chicken/Nigeria/OD9/06 AM503019
A/chicken/Nigeria/IF10/06 AM503024
A/chicken/Nigeria/957-20/06 CY016286
A/chicken/Nigeria/AB14/06 AM503025
A/chicken/Nigeria/AB13/06 AM503020
A/swan/Germany/R65/06 DQ464355
A/Cygnus olor/Croatia/1/05
2.2.3
A/chicken/Nigeria/228-10/06
79
A/chicken/Nigeria/SO452/06 AM262561
A/chicken/Nigeria/228-6/06
A/chicken/Nigeria/228-5/05
A/chicken/Nigeria/SO493/06 AM262562
A/chicken/Nigeria/SO300/06 AM262560
A/chicken/Nigeria/SO494/06 AM262563
100
A/chicken/Egypt/2253-1/06
A/swan/Slovenia/760/06 CY017045
A/turkey/Turkey/1/01 EF619973
A/Cygnus olor/Italy/808/06
A/goose/Hungary/3413/07 EF446781
A/chicken/Sudan/1784-8/06
A/chicken/Sudan/2115-10/06
A/chicken/BurkinaFaso/13.1/06
A/chicken/Nigeria/641/06 CY016278
A/chicken/IvoryCoast/1787-35/06
A/chicken/Kurgan/05/05 DQ449634
A/chicken/Crimea/08/05 DQ650665
2.2.2
A/Cygnus olor/Italy/742/06 CY017037
A/Cygnus cygnus/Iran/754/06
A/chicken/Moscow/2/07 EF474448
A/whooper swan/Mongolia/2/06
A/chicken/Afghanistan/1207/06
A/chickenAfghanistan/1573-47/06
2.2.1
A/duck/Korea/ESD1/03 AY676044
A/crow/Osaka/102/04 AB189063
A/crested eagle/Belgium/01/04
A/chicken/Thailand/PC168/06
100
A/duck/Parepare/BBVM/05 DQ493008
A/Indonesia/CDC1047/07 CY019426
A/China/GD01/06 DQ835315
A/munia/HongKong/2454/06
A/goose/Shantou/3295/06 EF124247
A/chicken/Hubei/wh/97 DQ997103
A/HongKong/486/97 AF084275
A/HongKong/542/97 AF102670
0.005 substitutions/site

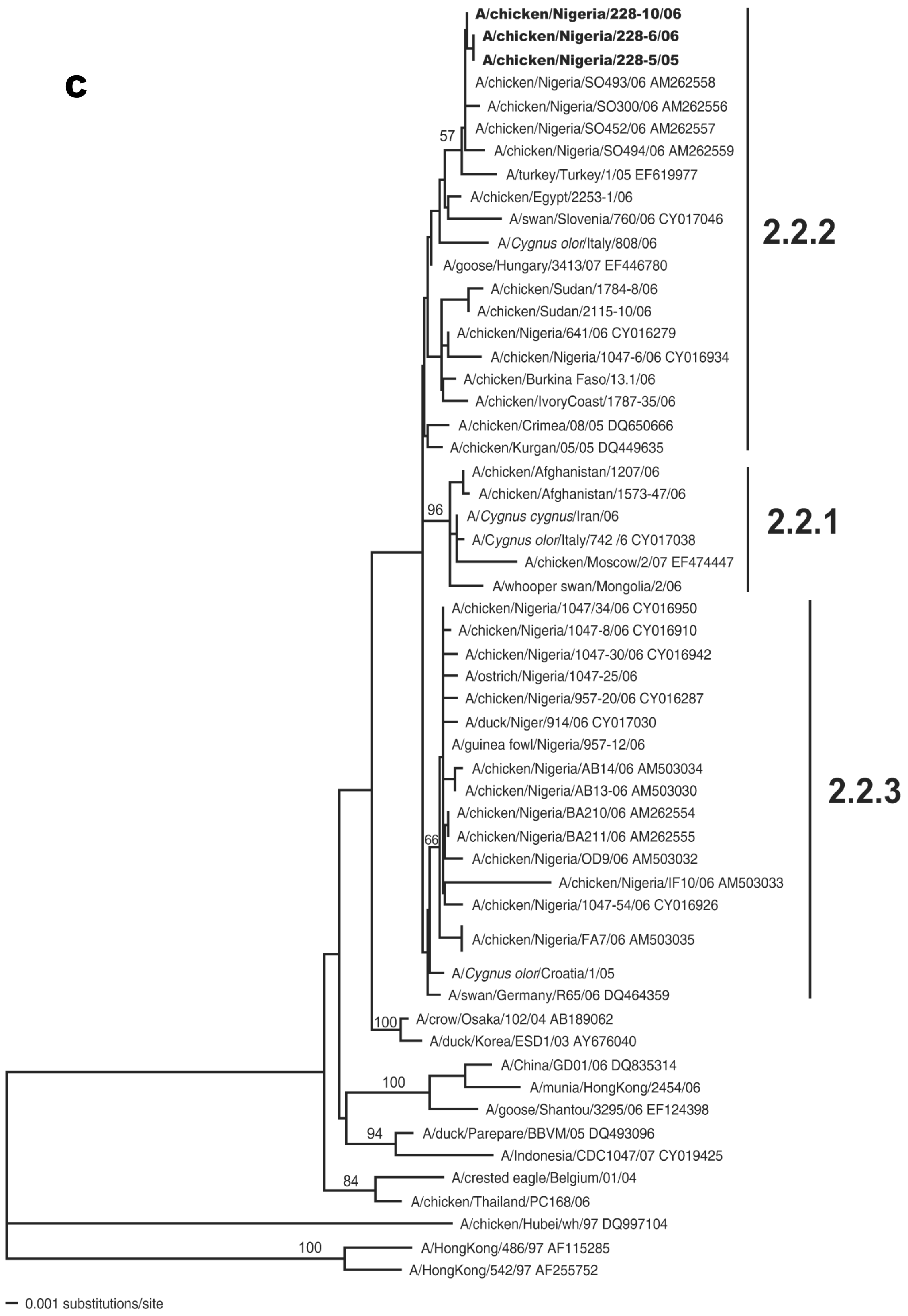
c
A/chicken/Nigeria/228-10/06
A/chicken/Nigeria/228-6/06
A/chicken/Nigeria/228-5/05
A/chicken/Nigeria/SO493/06 AM262558
A/chicken/Nigeria/SO300/06 AM262556
A/chicken/Nigeria/SO452/06 AM262557
A/chicken/Nigeria/SO494/06 AM262559
57
A/turkey/Turkey/1/05 EF619977
A/chicken/Egypt/2253-1/06
A/swan/Slovenia/760/06 CY017046
A/Cygnus olor/Italy/808/06
A/goose/Hungary/3413/07 EF446780
A/chicken/Sudan/1784-8/06
A/chicken/Sudan/2115-10/06
A/chicken/Nigeria/641/06 CY016279
A/chicken/Nigeria/1047-6/06 CY016934
A/chicken/Burkina Faso/13.1/06
A/chicken/IvoryCoast/1787-35/06
A/chicken/Crimea/08/05 DQ650666
A/chicken/Kurgan/05/05 DQ449635
2.2.2
A/chicken/Afghanistan/1207/06
A/chicken/Afghanistan/1573-47/06
96
A/Cygnus cygnus/Iran/06
A/Cygnus olor/Italy/742 /6 CY017038
A/chicken/Moscow/2/07 EF474447
A/whooper swan/Mongolia/2/06
2.2.1
A/chicken/Nigeria/1047/34/06 CY016950
A/chicken/Nigeria/1047-8/06 CY016910
A/chicken/Nigeria/1047-30/06 CY016942
A/ostrich/Nigeria/1047-25/06
A/chicken/Nigeria/957-20/06 CY016287
A/duck/Niger/914/06 CY017030
A/guinea fowl/Nigeria/957-12/06
A/chicken/Nigeria/AB14/06 AM503034
A/chicken/Nigeria/AB13-06 AM503030
A/chicken/Nigeria/BA210/06 AM262554
A/chicken/Nigeria/BA211/06 AM262555
66
A/chicken/Nigeria/OD9/06 AM503032
A/chicken/Nigeria/IF10/06 AM503033
A/chicken/Nigeria/1047-54/06 CY016926
A/chicken/Nigeria/FA7/06 AM503035
A/Cygnus olor/Croatia/1/05
A/swan/Germany/R65/06 DQ464359
2.2.3
100
A/crow/Osaka/102/04 AB189062
A/duck/Korea/ESD1/03 AY676040
A/China/GD01/06 DQ835314
100
A/munia/HongKong/2454/06
A/goose/Shantou/3295/06 EF124398
94
A/duck/Parepare/BBVM/05 DQ493096
A/Indonesia/CDC1047/07 CY019425
84
A/crested eagle/Belgium/01/04
A/chicken/Thailand/PC168/06
A/chicken/Hubei/wh/97 DQ997104
100
A/HongKong/486/97 AF115285
A/HongKong/542/97 AF255752
0.001 substitutions/site

D

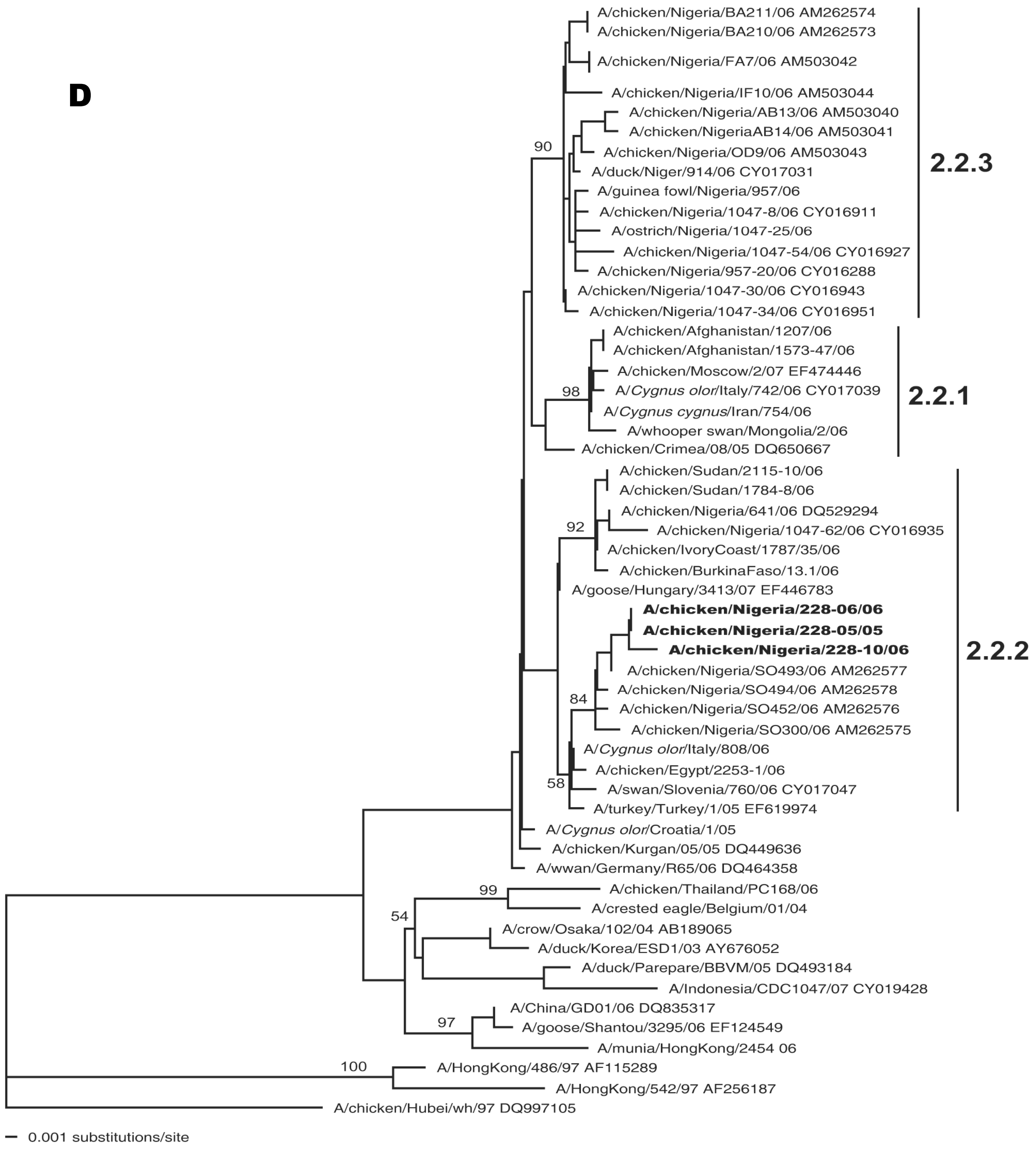
A/chicken/Nigeria/BA211/06 AM262574
A/chicken/Nigeria/BA210/06 AM262573
A/chicken/Nigeria/FA7/06 AM503042
A/chicken/Nigeria/IF10/06 AM503044
A/chicken/Nigeria/AB13/06 AM503040
A/chicken/NigeriaAB14/06 AM503041
A/chicken/Nigeria/OD9/06 AM503043
A/duck/Niger/914/06 CY017031
A/guinea fowl/Nigeria/957/06
A/chicken/Nigeria/1047-8/06 CY016911
A/ostrich/Nigeria/1047-25/06
A/chicken/Nigeria/1047-54/06 CY016927
A/chicken/Nigeria/957-20/06 CY016288
A/chicken/Nigeria/1047-30/06 CY016943
A/chicken/Nigeria/1047-34/06 CY016951
90
2.2.3
A/chicken/Afghanistan/1207/06
A/chicken/Afghanistan/1573-47/06
A/chicken/Moscow/2/07 EF474446
A/Cygnus olor/Italy/742/06 CY017039
A/Cygnus cygnus/Iran/754/06
A/whooper swan/Mongolia/2/06
A/chicken/Crimea/08/05 DQ650667
98
2.2.1
A/chicken/Sudan/2115-10/06
A/chicken/Sudan/1784-8/06
A/chicken/Nigeria/641/06 DQ529294
A/chicken/Nigeria/1047-62/06 CY016935
A/chicken/IvoryCoast/1787/35/06
A/chicken/BurkinaFaso/13.1/06
A/goose/Hungary/3413/07 EF446783
92
A/chicken/Nigeria/228-06/06
A/chicken/Nigeria/228-05/05
A/chicken/Nigeria/228-10/06
A/chicken/Nigeria/SO493/06 AM262577
A/chicken/Nigeria/SO494/06 AM262578
A/chicken/Nigeria/SO452/06 AM262576
A/chicken/Nigeria/SO300/06 AM262575
A/Cygnus olor/Italy/808/06
A/chicken/Egypt/2253-1/06
A/swan/Slovenia/760/06 CY017047
A/turkey/Turkey/1/05 EF619974
84
58
2.2.2
A/Cygnus olor/Croatia/1/05
A/chicken/Kurgan/05/05 DQ449636
A/wwan/Germany/R65/06 DQ464358
A/chicken/Thailand/PC168/06
A/crested eagle/Belgium/01/04
99
54
A/crow/Osaka/102/04 AB189065
A/duck/Korea/ESD1/03 AY676052
A/duck/Parepare/BBVM/05 DQ493184
A/Indonesia/CDC1047/07 CY019428
A/China/GD01/06 DQ835317
A/goose/Shantou/3295/06 EF124549
A/munia/HongKong/2454 06
97
A/HongKong/486/97 AF115289
A/HongKong/542/97 AF256187
100
A/chicken/Hubei/wh/97 DQ997105
0.001 substitutions/site
